# Supplementary material for: Multivariate analysis for agro-morphological and quality traits in groundnut (Arachis hypogaea L.) genotypes in Eastern Ethiopia
Source: PLoS One. 2026 Apr 30;21(4):e0347850. doi: 10.1371/journal.pone.0347850 (PMC13132432; doi:10.1371/journal.pone.0347850)
Supplement: S1 Table — (DOCX) [file pone.0347850.s001.docx]

|  | G2 | G3 | G4 | G5 | G6 | G7 | G8 | G9 | G10 | G11 | G12 | G13 | G14 | G15 | G16 |
| --- | --- | --- | --- | --- | --- | --- | --- | --- | --- | --- | --- | --- | --- | --- | --- |
| G1 | 5.1962 | 6.6332 | 4.1231 | 6.4807 | 5.9161 | 3.7417 | 5.4772 | 4.0000 | 3.7417 | 3.4641 | 5.0000 | 4.6904 | 5.0990 | 6.5574 | 5.1962 |
| G2 |  | 7.1414 | 6.4807 | 7.6811 | 7.6158 | 5.3852 | 4.3589 | 5.7446 | 5.3852 | 5.1962 | 7.0711 | 5.0000 | 5.0000 | 7.6158 | 7.3485 |
| G3 |  |  | 6.7082 | 6.6332 | 7.2801 | 5.8310 | 6.1644 | 6.3246 | 7.4833 | 4.8990 | 5.5678 | 6.1644 | 4.6904 | 6.0828 | 7.2801 |
| G4 |  |  |  | 6.4031 | 4.4721 | 5.0000 | 5.1962 | 3.8730 | 4.1231 | 3.8730 | 4.2426 | 4.7958 | 6.2450 | 6.7823 | 3.4641 |
| G5 |  |  |  |  | 5.7446 | 5.6569 | 6.4807 | 6.0000 | 6.3246 | 5.4772 | 4.3589 | 6.4807 | 4.6904 | 5.7446 | 6.4031 |
| G6 |  |  |  |  |  | 5.1962 | 5.7446 | 5.0000 | 5.9161 | 5.5678 | 4.4721 | 5.1962 | 6.8557 | 6.6332 | 4.4721 |
| G7 |  |  |  |  |  |  | 5.2915 | 4.8990 | 5.2915 | 3.4641 | 4.7958 | 5.0990 | 4.0000 | 6.7082 | 5.0000 |
| G8 |  |  |  |  |  |  |  | 4.0000 | 4.0000 | 4.8990 | 5.7446 | 4.0000 | 4.8990 | 6.2450 | 5.1962 |
| G9 |  |  |  |  |  |  |  |  | 2.8284 | 4.4721 | 4.7958 | 3.7417 | 4.8990 | 5.1962 | 3.3166 |
| G10 |  |  |  |  |  |  |  |  |  | 4.2426 | 5.7446 | 4.0000 | 5.8310 | 6.4031 | 4.5826 |
| G11 |  |  |  |  |  |  |  |  |  |  | 3.8730 | 4.0000 | 4.4721 | 6.5574 | 5.1962 |
| G12 |  |  |  |  |  |  |  |  |  |  |  | 4.7958 | 4.7958 | 5.8310 | 4.4721 |
| G13 |  |  |  |  |  |  |  |  |  |  |  |  | 5.4772 | 5.7446 | 5.5678 |
| G14 |  |  |  |  |  |  |  |  |  |  |  |  |  | 5.1962 | 6.0828 |
| G15 |  |  |  |  |  |  |  |  |  |  |  |  |  |  | 6.9282 |

Appendix Table 1. Euclidean distances of groundnut genotypes estimated from mean values of genotypes for 13 quantitative traits (n=36)

|  | Appendix Table 1. (Continued) | | | | | | | | | | |
| --- | --- | --- | --- | --- | --- | --- | --- | --- | --- | --- | --- |
|  | G17 | G18 | G19 | G20 | G21 | G22 | G23 | G24 | G25 | G26 | G27 |
| G1 | 6.0828 | 5.4772 | 5.6569 | 4.1231 | 4.6904 | 5.4772 | 5.4772 | 4.0000 | 5.7446 | 5.9161 | 3.4641 |
| G2 | 6.1644 | 6.4031 | 7.0000 | 5.6569 | 5.0000 | 7.1414 | 5.9161 | 4.3589 | 6.6332 | 5.6569 | 4.1231 |
| G3 | 8.0623 | 5.0990 | 4.0000 | 7.2801 | 6.7823 | 5.4772 | 8.4853 | 5.4772 | 6.8557 | 6.5574 | 6.9282 |
| G4 | 6.1644 | 5.3852 | 6.4031 | 5.4772 | 4.1231 | 6.8557 | 5.3852 | 4.3589 | 4.6904 | 6.1644 | 3.6056 |
| G5 | 8.0623 | 5.0990 | 5.0990 | 6.5574 | 5.0990 | 6.7823 | 6.6332 | 6.4807 | 6.4031 | 4.7958 | 6.3246 |
| G6 | 6.6332 | 4.7958 | 7.1414 | 5.4772 | 4.3589 | 7.9373 | 6.5574 | 4.7958 | 4.4721 | 5.4772 | 4.7958 |
| G7 | 7.4162 | 5.2915 | 5.0990 | 4.1231 | 4.4721 | 6.1644 | 6.4807 | 4.0000 | 5.3852 | 5.5678 | 3.7417 |
| G8 | 7.0000 | 4.2426 | 6.1644 | 6.2450 | 3.4641 | 7.3485 | 4.8990 | 3.7417 | 4.3589 | 5.7446 | 3.7417 |
| G9 | 6.5574 | 4.4721 | 6.0000 | 4.1231 | 3.7417 | 6.0000 | 3.4641 | 3.4641 | 4.7958 | 6.4031 | 3.1623 |
| G10 | 6.5574 | 4.8990 | 6.4807 | 5.3852 | 3.7417 | 6.7823 | 3.4641 | 4.4721 | 4.7958 | 6.5574 | 2.8284 |
| G11 | 5.7446 | 4.0000 | 3.7417 | 5.0000 | 4.4721 | 5.4772 | 5.8310 | 4.0000 | 4.7958 | 5.0000 | 3.7417 |
| G12 | 6.1644 | 4.1231 | 4.1231 | 5.4772 | 4.5826 | 5.7446 | 5.9161 | 4.5826 | 4.4721 | 4.6904 | 5.3852 |
| G13 | 4.3589 | 4.6904 | 6.0000 | 5.0000 | 4.2426 | 5.0990 | 4.2426 | 2.4495 | 3.6056 | 5.1962 | 3.7417 |
| G14 | 7.8102 | 5.0990 | 4.0000 | 5.0000 | 5.0990 | 5.2915 | 6.1644 | 4.6904 | 6.4031 | 5.1962 | 5.0990 |
| G15 | 8.3666 | 5.7446 | 6.8557 | 6.1644 | 6.7082 | 5.1962 | 6.5574 | 5.9161 | 6.0000 | 6.0000 | 6.2450 |
| G16 | 8.0000 | 5.1962 | 6.5574 | 5.4772 | 3.8730 | 7.8102 | 5.0000 | 4.7958 | 4.6904 | 7.3485 | 4.3589 |
| G17 |  | 6.8557 | 7.6811 | 6.4807 | 6.2450 | 6.7082 | 6.5574 | 5.0000 | 6.6332 | 5.6569 | 6.0828 |
| G18 |  |  | 4.2426 | 6.0828 | 4.4721 | 7.0711 | 6.1644 | 4.8990 | 4.3589 | 5.1962 | 4.8990 |
| G19 |  |  |  | 6.8557 | 6.0000 | 5.8310 | 7.6158 | 5.8310 | 6.5574 | 6.2450 | 6.4807 |
| G20 |  |  |  |  | 5.5678 | 6.2450 | 5.3852 | 4.3589 | 6.4807 | 5.6569 | 3.8730 |
| G21 |  |  |  |  |  | 7.0711 | 4.2426 | 3.4641 | 4.1231 | 5.3852 | 3.4641 |
| G22 |  |  |  |  |  |  | 6.9282 | 5.0990 | 6.8557 | 6.5574 | 6.7823 |
| G23 |  |  |  |  |  |  |  | 4.6904 | 5.1962 | 6.5574 | 4.4721 |
| G24 |  |  |  |  |  |  |  |  | 4.1231 | 5.1962 | 3.4641 |
| G25 |  |  |  |  |  |  |  |  |  | 5.6569 | 4.5826 |
| G26 |  |  |  |  |  |  |  |  |  |  | 5.5678 |

|  |  | | | | | | | | | |
| --- | --- | --- | --- | --- | --- | --- | --- | --- | --- | --- |
|  | G28 | G29 | G30 | G31 | G32 | G33 | G34 | G35 | G36 |  |
| G1 | 6.0000 | 6.0000 | 6.9282 | 4.7958 | 5.5678 | 5.7446 | 6.1644 | 5.2915 | 4.6904 |  |
| G2 | 6.4031 | 6.4031 | 7.8102 | 5.6569 | 6.4807 | 7.4833 | 7.0000 | 6.4031 | 5.7446 |  |
| G3 | 7.3485 | 6.9282 | 8.4853 | 5.3852 | 6.0828 | 7.5498 | 8.3666 | 5.2915 | 4.0000 |  |
| G4 | 5.7446 | 7.2801 | 6.8557 | 4.2426 | 4.6904 | 4.8990 | 5.0000 | 5.0000 | 5.1962 |  |
| G5 | 6.7823 | 4.8990 | 5.2915 | 5.0000 | 6.4031 | 6.2450 | 6.0000 | 3.7417 | 4.8990 |  |
| G6 | 5.9161 | 8.0623 | 6.2450 | 4.6904 | 5.4772 | 5.8310 | 3.8730 | 4.7958 | 5.7446 |  |
| G7 | 5.2915 | 6.7823 | 7.3485 | 3.8730 | 5.5678 | 6.4031 | 6.3246 | 4.2426 | 3.7417 |  |
| G8 | 3.7417 | 6.1644 | 6.9282 | 3.6056 | 3.6056 | 5.1962 | 5.6569 | 4.6904 | 5.2915 |  |
| G9 | 4.2426 | 5.6569 | 6.0000 | 4.1231 | 3.8730 | 3.3166 | 4.8990 | 4.4721 | 4.2426 |  |
| G10 | 4.8990 | 5.8310 | 6.1644 | 4.3589 | 3.8730 | 4.5826 | 6.0000 | 5.2915 | 5.8310 |  |
| G11 | 5.6569 | 6.0000 | 6.3246 | 3.3166 | 4.3589 | 5.7446 | 6.7823 | 4.2426 | 4.0000 |  |
| G12 | 5.3852 | 5.5678 | 5.3852 | 3.7417 | 5.2915 | 4.2426 | 5.0000 | 3.0000 | 3.8730 |  |
| G13 | 5.4772 | 6.1644 | 5.2915 | 4.7958 | 4.7958 | 5.1962 | 5.8310 | 4.0000 | 4.6904 |  |
| G14 | 5.4772 | 4.2426 | 7.0711 | 4.3589 | 5.7446 | 5.9161 | 6.6332 | 3.7417 | 2.4495 |  |
| G15 | 7.2801 | 4.7958 | 7.2801 | 6.1644 | 5.8310 | 6.3246 | 6.8557 | 4.3589 | 4.7958 |  |
| G16 | 3.6056 | 7.2801 | 7.0000 | 3.7417 | 4.4721 | 3.1623 | 4.5826 | 5.0000 | 5.3852 |  |
| G17 | 8.5440 | 7.9373 | 5.9161 | 7.3485 | 7.8740 | 7.6158 | 6.7082 | 6.8557 | 6.8557 |  |
| G18 | 4.6904 | 5.6569 | 6.1644 | 2.6458 | 3.0000 | 5.0000 | 6.1644 | 4.4721 | 5.0990 |  |
| G19 | 6.3246 | 5.6569 | 7.3485 | 4.3589 | 5.7446 | 6.2450 | 8.0000 | 4.6904 | 4.2426 |  |
| G20 | 6.2450 | 6.5574 | 6.5574 | 5.6569 | 6.3246 | 6.0000 | 5.7446 | 5.3852 | 4.1231 |  |
| G21 | 4.0000 | 6.0000 | 5.4772 | 3.3166 | 4.7958 | 4.5826 | 3.7417 | 4.2426 | 5.0990 |  |
| G22 | 8.3666 | 5.6569 | 7.0711 | 7.1414 | 7.6811 | 7.2801 | 8.1240 | 4.8990 | 4.2426 |  |
| G23 | 4.8990 | 5.4772 | 4.6904 | 5.5678 | 5.5678 | 3.8730 | 5.2915 | 5.2915 | 6.0000 |  |
| G24 | 4.8990 | 6.3246 | 6.0000 | 4.3589 | 5.1962 | 5.1962 | 4.8990 | 4.0000 | 3.7417 |  |
| G25 | 4.5826 | 6.7082 | 6.0828 | 3.7417 | 4.0000 | 4.8990 | 5.5678 | 3.8730 | 5.9161 |  |
| G26 | 7.1414 | 5.5678 | 5.1962 | 5.2915 | 6.4807 | 7.2111 | 5.7446 | 4.3589 | 5.0000 |  |
| G27 | 4.8990 | 6.4807 | 6.7823 | 3.8730 | 4.1231 | 5.3852 | 5.2915 | 4.8990 | 4.8990 |  |
| G28 |  | 6.6332 | 7.0711 | 3.3166 | 4.1231 | 3.6056 | 5.6569 | 5.0990 | 5.8310 |  |
| G29 |  |  | 6.0000 | 5.7446 | 6.4031 | 5.7446 | 7.0711 | 4.8990 | 5.2915 |  |
| G30 |  |  |  | 6.5574 | 7.2801 | 5.9161 | 5.8310 | 5.2915 | 6.4807 |  |
| G31 |  |  |  |  | 2.8284 | 4.4721 | 5.5678 | 3.8730 | 4.5826 |  |
| G32 |  |  |  |  |  | 4.6904 | 6.7082 | 5.0000 | 5.7446 |  |
| G33 |  |  |  |  |  |  | 5.0000 | 4.7958 | 5.5678 |  |
| G34 |  |  |  |  |  |  |  | 5.2915 | 6.0000 |  |
| G35 |  |  |  |  |  |  |  |  | 3.1623 |  |
