# Supplementary material for: Multivariate analysis for agro-morphological and quality traits in groundnut (Arachis hypogaea L.) genotypes in Eastern Ethiopia
Source: PLoS One. 2026 Apr 30;21(4):e0347850. doi: 10.1371/journal.pone.0347850 (PMC13132432; doi:10.1371/journal.pone.0347850)
Supplement: Attachment — Submitted filename: Arachis comments.docx [file pone.0347850.s003.docx]

The MS “Multivariate Analysis for Agro-Morphological and Quality Traits in Groundnut (Arachis hypogaea L.) Genotypes in Eastern Ethiopia” have following suggestions

1. Title

Ok

2. Abstract

Some redundancy (e.g., repeating “genotypes were grouped…”).

Missing clear concluding sentence with breeding implication.

“subjected for principal component” → subjected to principal component analysis

“numbers of genotypes” → number of genotypes

“Based on the result of manuscript” → unclear and incorrect

Follow strict structure: Background → Objective → Methods → Key Results → Conclusion.

End with clear breeding application.

Add one strong concluding line, e.g.:

The wide genetic divergence observed among selected genotypes provides valuable parental material for hybridization and genetic improvement of groundnut in Ethiopia.

3. Keywords

Missing region-specific or application-oriented terms.

4. Introduction

Several repetitions (importance of groundnut, nutrition).

Some irrelevant global statistics not directly linked to study aim.

Transition from global → Ethiopia → research gap is weak.

Suggested final paragraph revision:

Despite the importance of groundnut in eastern Ethiopia, comprehensive information on genetic divergence based on agro-morphological and quality traits remains limited. Therefore, this study aimed to assess genetic diversity among groundnut genotypes using multivariate statistical approaches to identify divergent and superior parental lines for future breeding programs.

5. Materials and Methods

5.1 Study Area

Strengths

Some values (1955–2015 averages) unnecessary.

5.2 Experimental Materials

Table formatting inconsistent.

“Breeding status” column could be simplified.

5.3 Experimental Design & Management

Inconsistency in season:

Earlier: 2023 post-rainy

Later: 2022 post-rainy

Spacing description confusing:

“0.6, 0.1, 0.5 m” unclear.

Correct season consistently.

6. Data Collection

Combine similar trait descriptions.

Ensure consistent abbreviations (DTM vs DPM).

7. Statistical Analysis

No justification for number of PCs retained (eigenvalue >1 rule mentioned later, not here).

No ANOVA reported prior to multivariate analysis.

Add:

Prior to multivariate analysis, analysis of variance (ANOVA) was conducted to assess the presence of significant variation among genotypes.

8. Results

8.1 Principal Component Analysis

Minor inconsistencies:

HSW vs HKW

NPPS typo

Correlation missing

If possible include correlation in results before pca

Follow reference paper <https://doi.org/10.1186/s12870-025-06335-x> and

https://doi.org/10.1038/s41598-024-81828-7

8.2 Cluster Analysis

Cluster numbering inconsistent (Cluster V vs VI confusion).

Consistent cluster numbering

8.3 Cluster Mean Analysis

“contained greater mean values” → showed higher mean values

Summarize major trends instead of describing each cluster in detail.

8.4 Genetic Distance

One typo:

“Gv10 and Gv27(8.83)” → incorrect (should be 2.83). Check

9. Discussion

Trait interrelationships

Link the key results with latest/recent findings

10. Conclusion (Missing / Weak)

Mention future research

12. References

Check years (FAOSTAT 2020 vs 2022 inconsistency).

Add some latest reference
