# Supplementary material for: Multivariate analysis for agro-morphological and quality traits in groundnut (Arachis hypogaea L.) genotypes in Eastern Ethiopia
Source: PLoS One. 2026 Apr 30;21(4):e0347850. doi: 10.1371/journal.pone.0347850 (PMC13132432; doi:10.1371/journal.pone.0347850)
Supplement: Attachment — Submitted filename: Response to Reviewers.docx [file pone.0347850.s005.docx]

| **Reviewer Comment** | **Author Response** |
| --- | --- |
| Please amend your list of authors on the manuscript to ensure that each author is linked to an affiliation. | Thank you for your valuable comment. We have revised the author list to ensure that each author is clearly linked to the appropriate affiliation, following the formatting guidelines of PLOS ONE. All authors are affiliated with the same institution, and this has now been indicated using a shared affiliation number.  The updated author and affiliation details are presented as follows:  **Desu Beriso¹*, Seltene Abady¹, Abdi Mohammed¹**  **¹ Department of Agriculture, Haramaya University, Haramaya, Ethiopia**  ***Corresponding author: desuberiso500@gmail.com**  These changes have been incorporated into the revised manuscript. |
| **Title:** OK | We appreciate the positive evaluation. No changes were made to the title. |
| **Abstract:** Redundancy (e.g., repeating “genotypes were grouped…”). | The abstract has been carefully revised to remove redundant statements and improve conciseness. |
| **Abstract:** Missing clear concluding sentence with breeding implication. | A strong concluding statement has been added: *“Therefore, the genetically divergent genotypes identified in this study provide valuable parental material for hybridization, offering strong potential for the development of improved groundnut varieties adapted to Ethiopian agro-ecologies.”* |
| **Abstract**: “subjected for principal component” → grammatical correction needed. | Revised to “subjected to principal component analysis.” |
| **Abstract:** “numbers of genotypes” → incorrect grammar. | Corrected to “number of genotypes.” |
| **Abstract:** “Based on the result of manuscript” unclear. | The sentence was rewritten for clarity and scientific precision. |
| **Abstract:** Follow strict structure (Background → Objective → Methods → Results → Conclusion). | The abstract has been restructured accordingly to follow the suggested format. |
| **Keywords:** Missing region-specific or application-oriented terms. | Keywords were revised to include: Eastern Ethiopia, Genetic divergence, Groundnut breeding, Multivariate analysis, Principal component analysis, Cluster analysis. |
| **Introduction:** Repetition regarding importance of groundnut. | Repetitive statements were removed and content streamlined. |
| **Introduction:** Some global statistics not directly linked to study aim. | Unnecessary global statistics were reduced to maintain focus on the study objective. |
| **Introduction:** Weak transition from global → Ethiopia → research gap. | The introduction was reorganized to clearly present global importance, Ethiopian context, knowledge gap, and study objective. |
| **Introduction:** Suggested revised final paragraph. | The final paragraph was revised as suggested to clearly state the research gap and objective. |
| **Materials & Methods, Study Area**: 1955–2015 averages unnecessary. | Long-term averages were removed; only relevant experimental season data were retained. |
| **Experimental Materials:** Table formatting inconsistent. | Table formatting was standardized throughout the manuscript. |
| **Experimental Materials:** “Breeding status” column could be simplified. | Simplified into clear categories Advanced line. |
| **Experimental Design:** Season inconsistency (2022 vs 2023). | Corrected consistently to “2023 post-rainy season” throughout the manuscript. |
| **Experimental Design:** Spacing description unclear (0.6, 0.1, 0.5 m). | Clarified as: 0.6 m between rows, 0.1 m between plants, and 0.5 m between plots. |
| **Data Collection**: Combine similar trait descriptions. | Traits were grouped under phenological, growth traits, yield-related traits, yield, and derived yield trait for clarity. |
| **Data Collection:** Inconsistent abbreviations (DTM vs DPM). | Abbreviations standardized consistently throughout (DF75, DM90, HKW, etc.). |
| **Statistical Analysis:** No ANOVA reported prior to multivariate analysis. | Added statement: “Prior to multivariate analysis, analysis of variance (ANOVA) was conducted to determine the presence of significant variation among genotypes.” |
| **Statistical Analysis**: No justification for number of PCs retained. | Added justification: “Principal components with eigenvalues greater than 1.0 were retained following Kaiser’s criterion.” |
| **Results** – PCA: Inconsistencies (HSW vs HKW). | Standardized to HKW throughout the manuscript. |
| **Results** – PCA: NPPS typo. | Typographical error corrected to NPP. |
| **Results** – Correlation missing before PCA. | Correlation matrix analysis was added prior to PCA to describe trait interrelationships. |
| **Results** – Follow recent reference papers. | Interpretation of PCA and clustering was aligned with recent multivariate studies (2024–2025). |
| **Cluster Analysis:** Cluster numbering inconsistent. | Cluster numbering corrected consistently (Cluster I–VI). |
| **Cluster Mean Analysis**: “Contained greater mean values” wording. | Revised to “showed higher mean values.” |
| **Cluster Mean Analysis**: Overly descriptive. | Results were summarized to highlight major trends rather than describing each cluster in detail. |
| **Genetic Distance:** Typo in Eucledean Distance (ED) value (8.83 vs 2.83). | Corrected after rechecking ED results. |
| **Discussion:** Trait interrelationships insufficiently explained. | Discussion expanded to explain correlations among yield and quality traits and their breeding implications. |
| **Discussion:** Link findings with latest research. | Recent (2024–2025) peer-reviewed studies were incorporated to strengthen discussion. |
| **Conclusion:** Weak and missing future research direction. | Conclusion rewritten to emphasize key findings, breeding implications, and future integration of molecular markers. |
| **References:** FAOSTAT year inconsistency. | Standardized citation year consistently throughout the manuscript. |
| **References:** Add latest references. | Recent literatures (2023–2025) were added and reference list updated according to journal format. |

**Final Statement to the Editor**

We sincerely thank the reviewer for the constructive and insightful comments. All suggestions have been carefully addressed, and the manuscript has been substantially improved in clarity, structure, and scientific rigor.
